# Hereditary Hemolytic Spherocytosis in the Active-Duty Population: A Unique Case

**DOI:** 10.7759/cureus.109278

**Published:** 2026-05-20

**Authors:** David Ahn, Jeffrey Berenberg

**Affiliations:** 1 Department of Internal Medicine, Tripler Army Medical Center, Honolulu, USA; 2 Department of Hematology and Oncology, Tripler Army Medical Center, Honolulu, USA

**Keywords:** adult-onset, genotype-phenotype correlation, hereditary spherocytosis, novel mutation, sptb gene

## Abstract

Hereditary spherocytosis (HS) is a common inherited cause of hemolytic anemia and is most often diagnosed in childhood. While the condition does not by itself disqualify affected adults from military service, its autosomal dominant inheritance has important implications for offspring, who may develop more severe disease. We report the case of a 38-year-old active-duty US Army soldier of Saipanese (Chamorro) descent who presented with persistent, unexplained fatigue -- most pronounced during strenuous physical activity -- despite adequate sleep. He had no formal family history of anemia, although one of his three children carries an undiagnosed anemia. Physical examination revealed splenomegaly. Laboratory evaluation demonstrated compensated hemolysis, as evidenced by a low-normal hemoglobin maintained by a reticulocyte response of 9.36%, a normal mean corpuscular volume, an elevated mean corpuscular hemoglobin concentration of 36.7 g/dL -- a characteristic diagnostic feature of HS -- an elevated red cell distribution width, undetectable haptoglobin, and elevated lactate dehydrogenase and indirect bilirubin. The peripheral blood smear was reported by the staff pathologist as a normocytic, normochromic anemia with occasional spherocyte-like forms and mild polychromasia. The patient also demonstrated mild-to-moderate hepatic iron overload despite the absence of transfusions. Genetic testing identified a heterozygous nonsense mutation in the *SPTB* gene, c.2175G>A (p.Trp725Ter), introducing a premature termination codon within exon 12 and consistent with HS type II. A concurrent pyruvate kinase variant of uncertain significance was also identified. The variant identified in our patient was subsequently deposited in ClinVar by the clinical testing laboratory (Variation ID: 2428713; classified pathogenic) and is absent from gnomAD v4.1.1; to our knowledge, this variant has not previously been described in the published clinical literature. This report provides the clinical and phenotypic characterization of the patient whose testing contributed to that ClinVar submission, and highlights the value of genetic evaluation in adults with unexplained hemolysis, particularly when the offspring is also affected.

## Introduction

Hereditary spherocytosis (HS) is a heterogeneous group of hemolytic anemias caused by mutations in genes encoding red blood cell (RBC) membrane proteins, most commonly *ANK1*, *SLC4A1*, and *SPTB*. While HS is the most prevalent non-immune hemolytic anemia in individuals of Northern European descent, its genetic landscape and clinical presentation in other ethnic populations remain less characterized [1]. The condition typically presents with a clinical triad of anemia, jaundice, and splenomegaly, although adult presentations are often masked by compensatory erythropoiesis [2].

The *SPTB* gene, located on chromosome 14q23.3, encodes the beta-spectrin protein, which is essential for maintaining the structural integrity and elasticity of the RBC membrane [3]. Mutations in *SPTB* typically follow an autosomal dominant inheritance pattern and account for approximately 15%-30% of HS cases worldwide [4].

We present the case of a 38-year-old active-duty US Army soldier of Saipanese (Chamorro) descent whose evaluation for persistent exertional fatigue led to the identification of a heterozygous nonsense mutation in the *SPTB* gene (c.2175G>A (p.Trp725Ter)). The variant identified in our patient was subsequently deposited in ClinVar by the clinical testing laboratory (ARUP Laboratories; Variation ID: 2428713; classification: pathogenic) [5], and is absent from gnomAD v4.1.1. To our knowledge, no clinical case report describing a patient with this variant has previously been published. Several features make this case instructive beyond the variant itself: an adult presentation with compensated hemolysis rather than overt anemia, mild-to-moderate hepatic iron overload despite the absence of transfusions, and a concurrent pyruvate kinase variant of uncertain significance. The case also has practical relevance to military medicine, in which the physical demands of training and deployment may bring otherwise compensated hemolytic states to clinical attention; medical fitness for retention is governed in the US Army by AR 40-501 [6].

## Case presentation

History of present illness

A 38-year-old male of Saipanese (Chamorro) descent, on active duty in the US Army, presented with an 11-year history of persistent, unexplained fatigue. Previously attributed to occupational and familial stressors, the patient recently noted a significant decline in performance during strenuous physical activities, including the Army Combat Fitness Test and ruck marches. He denied a formal family history of anemia, although one of his three children carries an undiagnosed anemia.

Diagnostic evaluation

Physical examination was significant for palpable splenomegaly. Initial laboratory studies (Table 1) demonstrated compensated hemolysis: a low-normal hemoglobin maintained by a brisk reticulocyte response of 9.36%, a normal mean corpuscular volume (MCV), an elevated mean corpuscular hemoglobin concentration (MCHC) of 36.7 g/dL -- a characteristic diagnostic feature of HS that reflects red cell dehydration and is one of the most useful automated screening parameters for the condition [1,7] -- and an elevated red cell distribution width (RDW) of 17.1%. Hemolytic markers included an elevated lactate dehydrogenase of 581 U/L, an indirect hyperbilirubinemia, and an undetectable haptoglobin. A direct antiglobulin test was negative, supporting a non-immune process. The peripheral blood smear was reviewed by the staff pathologist and reported as a normocytic, normochromic anemia with occasional spherocyte-like forms and mild polychromasia. In the context of elevated MCHC, elevated RDW, reticulocytosis, undetectable haptoglobin, indirect hyperbilirubinemia, and the *SPTB* variant identified on subsequent genetic testing (see below), these findings are collectively consistent with HS.

**Table 1:** Hematological and hemolytic profile at initial presentation Laboratory evaluation demonstrates compensated hemolysis with an elevated MCHC and markers of chronic hemolysis, including undetectable haptoglobin and elevated LDH. CBC, complete blood count; DAT, direct antiglobulin test; HS, hereditary spherocytosis; LDH, lactate dehydrogenase; MCHC, mean corpuscular hemoglobin concentration; MCV, mean corpuscular volume; RDW, red cell distribution width.

Category	Lab test	Patient result	Unit	Reference range	Interpretation
CBC	Hemoglobin	13.7	g/dL	13.5-17.5	Low-normal
CBC	Hematocrit	37.7	%	41.0-50.0	Low
CBC	MCV	90.0	fL	80.0-100.0	Normal
CBC	MCHC	36.3	g/dL	32.0-36.0	High (classic HS)
CBC	RDW	17.8	%	11.0-15.0	High
Hemolysis	Reticulocyte	9.0	%	0.5-1.5	High (compensatory)
Hemolysis	Haptoglobin	<10.0	mg/dL	30.0-200.0	Undetectable
Hemolysis	Total bilirubin	3.2	mg/dL	0.1-1.2	High (indirect)
Hemolysis	LDH	581.0	U/L	140.0-280.0	High
Hemolysis	Direct Coombs (DAT)	Negative	-	Negative	Non-immune

Standard membrane studies (EMA-binding flow cytometry and osmotic fragility testing) were not performed before genetic testing. Comprehensive genetic panel sequencing was selected on the basis of assay availability at our institution and the diagnostic value of identifying coexisting variants in a patient with atypical features and a positive family history of unexplained anemia in offspring. The absence of these confirmatory studies is acknowledged as a limitation of our diagnostic approach.

Imaging and secondary considerations

Abdominal ultrasound and 2D shear wave elastography confirmed splenomegaly with a splenic index of 592.75 and identified cholelithiasis without evidence of acute cholecystitis. Liver stiffness was measured at 1.30 m/sec (~5.07 kPa), at the upper limit of normal for hepatic fibrosis.

Given a significantly elevated transferrin saturation of 69% and a ferritin of 781.95 ng/mL in a non-transfused patient, further evaluation for iron overload was performed. A T2-weighted MRI protocol for quantitative iron evaluation revealed a liver iron concentration of 3.50 mg/g dry weight (reference: <2.0 mg/g) (Table 2), confirming mild-to-moderate hepatic iron overload. Iron overload in a non-transfused patient with HS most likely reflects chronic ineffective erythropoiesis with relative hepcidin suppression and increased gastrointestinal iron absorption -- a mechanism well described in chronic hemolytic states with an expanded erythroid compartment [8] -- with possible additive contributions from the concurrent pyruvate kinase variant and from a coexisting *HFE* variant. The patient was referred to medical genetics for *HFE* evaluation but was lost to follow-up. 

**Table 2:** Assessment of secondary complications and quantitative imaging Evaluation of systemic iron status and end-organ involvement reveals significant non-transfusional iron overload (elevated TSAT and MRI-LIC), palpable splenomegaly (increased splenic index), and cholelithiasis. LIC, liver iron concentration; MRI, magnetic resonance imaging; SWE, shear wave elastography; TSAT, transferrin saturation.

Domain	Diagnostic metric	Patient result	Unit	Normal range	Clinical significance
Iron	TSAT	69.0	%	15.0-50.0	Systemic overload
Iron	Ferritin	781.9	ng/mL	30.0-400.0	Elevated stores
Iron	LIC	3.5	mg/g DW	<2.0	Hepatic deposition
Imaging	Splenic index	592.7	-	<480.0	Splenomegaly
Imaging	Median liver stiffness (SWE)	1.30	m/sec	<1.35	Normal/upper limit
Imaging	Cholelithiasis	Present	-	Absent	Pigment gallstones
Metabolic	Folate	5.4	ng/mL	>5.9	Low-normal

Genetic analysis and management

Genetic testing using a hereditary hemolytic anemia panel identified a heterozygous nonsense mutation in the *SPTB* gene (c.2175G>A (p.Trp725Ter)), consistent with HS type II. The variant identified in our patient was subsequently deposited in ClinVar by the clinical testing laboratory (Variation ID: 2428713; classification: pathogenic; one-star review status) [5]. A concurrent variant in the* PKLR* gene encoding pyruvate kinase was also identified and classified as a variant of uncertain significance per American College of Medical Genetics and Genomics (ACMG) criteria.

The patient was started on folic acid supplementation. Given evidence of hepatic iron overload and the risk of biliary complications, he was referred to medical genetics to evaluate for possible co-inherited *HFE* mutations. He was monitored for worsening hemolysis, development of symptomatic gallstones, and parvovirus B19-associated red cell aplasia [9]. Intermittent low-volume phlebotomy was initiated to maintain ferritin below 200 ng/mL. Phlebotomy was selected over iron chelation given the mild-to-moderate degree of iron loading and patient preference, and is performed with extended intervals between sessions, including a hemoglobin floor of 12.5 g/dL, low-volume removals per session with serial monitoring of hemoglobin, ferritin, and clinical symptoms.

## Discussion

In our patient, the diagnosis was supported by identifying a heterozygous nonsense mutation in the *SPTB* gene (c.2175G>A (p.Trp725Ter)). This single-nucleotide G>A substitution converts the tryptophan codon TGG to the stop codon TGA, introducing a premature termination codon. The variant is expected to result in a truncated, non-functional beta-spectrin protein, with the variant transcript likely subject to nonsense-mediated mRNA decay [10]. Beta-spectrin plays a key role in maintaining the structural integrity of the RBC membrane, and defects in this protein weaken membrane stability. As a result, RBCs lose surface area, become more spherical, and are less deformable. These spherocytes are then removed by the spleen, which is the underlying mechanism of hemolysis in HS [11,12].

The c.2175G>A (p.Trp725Ter) variant identified in our patient was subsequently deposited in ClinVar by the clinical testing laboratory (Variation ID: 2428713) [5]. The submission classifies the variant as pathogenic on the basis of ACMG criteria, citing its absence from gnomAD v4.1.1 and the predicted molecular consequence of an early termination codon with likely nonsense-mediated decay. To our knowledge, no clinical case report describing the phenotype of a patient with this variant has previously been published; the present report therefore provides the clinical and phenotypic characterization of the patient whose testing contributed to the ClinVar submission. While other *SPTB* truncating mutations have been described, most reports involve different exons and are often identified in neonatal cases [3,13]. This case adds clinical and phenotypic data to a variant for which only molecular data were previously publicly available.

Atypical feature: non-transfusional iron overload

The most notable atypical feature in our patient is mild-to-moderate hepatic iron overload in the absence of any transfusion history. This is best explained by chronic ineffective erythropoiesis with relative hepcidin suppression and increased gastrointestinal iron absorption, a mechanism described across a spectrum of chronic hemolytic and dyserythropoietic anemias [8], with possible additive contributions from the concurrent pyruvate kinase variant of uncertain significance -- pyruvate kinase deficiency itself can contribute to ineffective erythropoiesis and iron homeostasis dysregulation [8] -- and from a coexisting *HFE* variant under evaluation. Iron overload is not commonly emphasized as a feature of HS in adult patients, and its presence in this case adds to the clinical characterization of the variant and underscores the importance of evaluating iron status in adults with chronic compensated hemolysis.

Complications of HS

Beyond the fatigue and exercise intolerance that prompted our patient’s presentation, HS is associated with several clinically important complications. Pigment gallstones develop in approximately half of untreated patients by the second to fifth decades of life as a consequence of chronic hemolysis and the resulting bilirubin load [1,2]; our patient already has cholelithiasis on imaging. Aplastic crisis in the setting of parvovirus B19 infection is a recognized acute complication, reflecting the virus’s tropism for erythroid progenitors superimposed on a shortened red cell lifespan [9]. Long-standing chronic hemolysis can also produce leg ulcers, gout, chronic dermatitis, and masses arising from extramedullary hematopoiesis [2]. Splenectomy remains the most effective intervention for symptomatic disease, with reliable improvement in hemoglobin and reduction in hemolysis; incomplete responses should prompt evaluation for accessory spleens, and partial or subtotal splenectomy has been investigated as a means of reducing post-splenectomy infection risk while preserving splenic immune function [1,14]. Where total splenectomy is performed, post-splenectomy infection prophylaxis and appropriate vaccination remain essential.

Implications for military service

In the active-duty population, compensated hemolytic states such as that observed in our patient may become clinically apparent only with the increased physical demands of training and deployment. Under US Army medical fitness standards (AR 40-501), HS does not by itself preclude continued service in the absence of severe anemia, transfusion dependence, or significant splenectomy-related complications [6], but the diagnosis has implications for activity tolerance, deployability to settings with limited medical support, and family planning. The autosomal-dominant inheritance pattern is particularly relevant given that one of our patients’ children carries an undiagnosed anemia, and cascade family evaluation is in progress.

## Conclusions

We have presented the case of a 38-year-old active-duty US Army soldier of Saipanese (Chamorro) descent with HS associated with a heterozygous nonsense mutation in the *SPTB* gene (c.2175G>A (p.Trp725Ter)). The variant identified in our patient has been deposited in ClinVar (Variation ID: 2428713) by the clinical testing laboratory and classified pathogenic; to our knowledge, no clinical case report describing a patient with this variant has previously been published, and this report provides the clinical and phenotypic characterization of the patient whose testing contributed to that submission. The atypical feature of mild-to-moderate hepatic iron overload in a non-transfused patient, together with a concurrent pyruvate kinase variant of uncertain significance, underscores the diagnostic complexity of chronic hemolysis in adults, particularly in non-Northern European populations. Although broad screening or management recommendations cannot be derived from a single case, this report supports consideration of comprehensive genetic testing in the workup of unexplained compensated hemolysis, particularly when offspring are affected, and underscores the importance of cascade family evaluation. Further reports and registry-level data will be necessary to establish the phenotypic spectrum and management implications of this and similar variants.
